# Problem Management Plus (PM+) for common mental disorders in a humanitarian setting in Pakistan; study protocol for a randomised controlled trial (RCT)

**DOI:** 10.1186/s12888-015-0602-y

**Published:** 2015-10-01

**Authors:** Marit Sijbrandij, Saeed Farooq, Richard A. Bryant, Katie Dawson, Syed Usman Hamdani, Anna Chiumento, Fareed Minhas, Khalid Saeed, Atif Rahman, Mark van Ommeren

**Affiliations:** Department of Clinical Psychology, VU University Amsterdam and EMGO Institute for Health and Care Research, Amsterdam, The Netherlands; Post Graduate Medical Institute, Lady Reading Hospital, Peshawar, Pakistan; School of Psychology, University of New South Wales, Sydney, Australia; University of Liverpool and Human Development Research Foundation, Islamabad, Pakistan; WHO Collaborating Centre for Mental Health Research, Training and Substance Abuse and Institute of Psychiatry, Rawalpindi Medical College, University of Health Sciences, Rawalpindi, Pakistan; WHO Regional Office for the Eastern Mediterranean, Cairo, Egypt; Department of Mental Health and Substance Abuse, World Health Organization (WHO), Geneva, Switzerland

## Abstract

**Background:**

In humanitarian settings common mental disorders (depression, anxiety disorders, posttraumatic stress disorder) are highly prevalent. The World Health Organization (WHO) has developed Problem Management Plus (PM+), a 5-session, individual psychological intervention program, delivered by paraprofessionals that addresses common mental disorders in people in communities affected by adversity. The objectives of this study are to test effectiveness and cost-effectiveness of the locally adapted PM+ compared to Treatment as usual (TAU) in Peshawar District, Pakistan.

**Methods:**

A randomised controlled trial will be conducted in 346 primary care attendees in 3 health care centres in Peshawar District, Pakistan. After informed consent, primary care attendees with high levels of psychological distress according to the General Health Questionnaire-12 (GHQ-12) and functional impairment (WHO Disability Assessment Schedule 2.0 (WHODAS)) will be assigned to PM+ (n = 173) or TAU (n = 173). At baseline, 1 week and 3 months following PM+, independent assessors will assess psychological distress with the Hospital Anxiety and Depression Scale (HADS), and functional disability with the WHODAS. Secondary outcomes are posttraumatic stress disorder (PTSD) symptoms, and client-perceived priority problems. Further, cost-effectiveness will be assessed using the Service Receipt Inventory (SRI).

**Discussion:**

If proven effective, PM+ will be rolled out to other areas for further adaptation and testing in diverse humanitarian settings.

**Trial registration:**

ACTRN12614001235695. Registered 26 November 2014. Australian New Zealand Clinical Trials Registry

## Background

Over the past 3 decades the north-western Pakistani province of Khyber Pakhtunkhwa, with Peshawar as its capital, has been affected by political instability, economic uncertainty, regional conflict, and continuous daily violence [1]. In such settings, common mental disorders are highly prevalent [2]. In Pakistan alone, the estimated prevalence of common mental disorders ranges between 10 and 16 % [3] and major depression is one of the major causes of disability [4]. These mental health conditions impair basic functioning required for survival and livelihoods. Accordingly, there is a great need for effective psychosocial interventions that can reduce symptoms of common mental disorders and improve daily functioning.

A major obstacle to addressing the mental health challenges in many humanitarian settings is that they occur in low and middle income countries (LMICs). LMICs typically lack sufficient numbers of specialized mental health care professionals, have poor systems to deliver evidence-based interventions, and cannot afford costs associated with intensive treatment programs [5]. Although psychological interventions, such as cognitive behavioural therapy (CBT), are effective in the treatment of common mental disorders that are evident in the wake of adversity [6, 7], these typically require expert mental health professionals providing treatments that are usually lengthy and costly to the health service. Overall, there is little evidence for treating mental health conditions in humanitarian settings [2], and the trials that have been conducted have focused on treatments for posttraumatic stress disorder (PTSD) [2]. Limitations of this approach are that PTSD treatments are usually relatively intensive and lengthy, and individuals affected by adversity and trauma typically have a range of other problems than PTSD, including depression and anxiety [2].

For interventions to be scalable in LMIC settings, they should be of short duration and sufficiently simple that they can be carried out by lay people in the community [8], and they should address a broad range of mental and psychosocial health problems relevant to communities affected by adversity. To meet these demands, WHO has developed a low-intensity 5-sessions program termed Problem Management Plus (PM+) which may be delivered by trained lay people [9]. This program is aimed at reducing symptoms of depression, anxiety, PTSD, stress in the wake of adversity and trauma. It comprises evidence-based techniques: of (a) problem solving, (b) stress management, (c) behavioural activation, and (d) accessing social support. Although developed following considerable international consultation, the WHO has adopted the position that implementation of the protocol should not occur until it has been proven to be effective via controlled trials in LMICs. To this end, this paper provides an overview of the trial protocol for the initial study of PM+ in the Peshawar District. The PM+ manual has been translated into Urdu and adapted to the local culture in the Peshawar District. Qualitative methods including free listing and key informant interviews [10] identified priority problems and health concepts from local perspectives which were used to inform intervention adaptation. Subsequent to this process, this study aims to (a) evaluate the effectiveness of the locally adapted version of PM+ in Peshawar in reducing symptoms of common mental disorders; and (b) evaluate the cost-effectiveness of PM+ in this setting.

## Methods

### Design

The study is a randomised controlled trial (RCT) that compares PM+ to treatment as usual (TAU) in primary care attendees in three primary care centres in Peshawar District, Pakistan. The primary outcomes are psychological distress in terms of states of anxiety and depression at 20 weeks after inclusion. Secondary outcomes are functional disability, PTSD symptoms, and cost-effectiveness.

### Participants

Participants will include primary care attendees who fulfil the following inclusion criteria: (a) score above 2 on a screening questionnaire for common mental disorders (General Health Questionnaire-21; GHQ-12) [11, 12] and (b) score above 16 on a screening questionnaire for functional impairments (WHO Disability Assessment Schedule 2.0; WHODAS) [13].

Exclusion criteria are: (a) suicide risk as defined in the mhGAP Intervention Guide [14]; (b) individuals with a severe mental disorder (e.g., psychotic disorders, substance dependence) or severe cognitive impairment (e.g., severe intellectual disability or dementia), based on definitions in the mhGAP Intervention Guide [14].

### Procedure

**Fig. 1:**
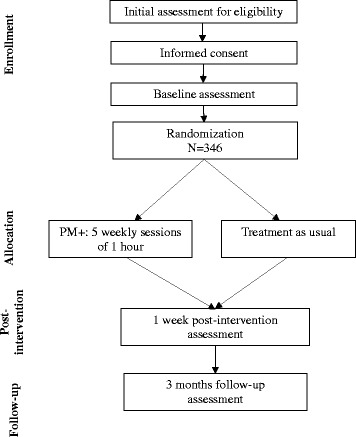
Flow diagram

The study will be carried out in three peri-urban primary healthcare centres (PHCs) in Peshawar District in Pakistan, overseen by staff of Lady Reading Hospital. All research assessments and PM+ sessions are conducted at the PHCs. The study flow diagram is presented in Fig. 1.

Potential participants are informed about the study. Informed consent entails a two-step procedure: 1. Informed consent for screening and 2. Informed consent for taking part in the PM+ trial. The latter is only required for participants meeting inclusion criteria. For each step respondents who decide to participate will be asked to complete a written consent form. For participants who are illiterate, witnessed oral consent and a thumb print in lieu of a signature will be asked, in line with recommendations from WHO [15]. The witness will be any staff member of at the PHC (except the medical officer or health care professional involved directly in the care of the patient in order to avoid potential explicit or implicit coercion); or any adult person not related to the participant who the participant is comfortable having present during consent.

Following informed consent for screening, a research assistant will record demographic characteristics and participants will be administered the GHQ-12, the WHODAS, and the suicide screening questions. If they are not at imminent risk of suicide, do not meet other exclusion criteria, and score above both the cut-offs of the GHQ-12 and the WHODAS, they will be provided informed consent information about the RCT. At least 24 h later, a research assistant will conduct the pre-trial assessment, which will comprise the Hospital Anxiety and Depression Scale (HADS) [16, 17], the Patient Health Questionnaire (PHQ-9) [18], the Harvard Trauma Questionnaire (HTQ) [19] events list, Life Events List for Pakistan (LELFP) [20], the PTSD Checklist for DSM-5 (PCL-5) [21], the Psychological Outcome Profiles instrument (PSYCHLOPS) [22] and the Service Receipt Inventory (SRI) [23].

If participants are not selected because they score below the cut-offs for the GHQ-12 or the WHODAS, they will be provided feedback on their test outcomes and reasons why they are not eligible for the study will be explained to them.

The post-intervention assessment (WHODAS, HADS, PHQ-9, PCL-5, HTQ events list, LELFP, PSYCHLOPS) will be scheduled 7 weeks after the pre-intervention assessment (i.e., 1 week after the 5^th^ PM+ session), and the follow-up assessment (WHODAS, HADS, PHQ-9, PCL-5, HTQ events list, LELFP, PSYCHLOPS, SRI) will be scheduled at 13 weeks after the post-intervention assessment (i.e., 20 weeks after inclusion, in line with the timing of the follow-up assessment for the PM+ participants). The participants who receive PM+ will also be administered the PSYCHLOPS by the lay-counsellor at the beginning of each PM+ session.

All assessments will be delivered in interview format as many participants are expected to be illiterate. All instruments will be administered by independent assessors blind to the allocation status of the participants. All assessors have received a 2-day training covering administering the instruments, common mental disorders, general interview techniques, and ethical research conduct. Ongoing monitoring of assessors’ competency will be conducted through regular supervision by the trial manager.

### Sample size

A total number of 346 participants will be included in this study. Since we are not aware of similar intervention studies that have been carried out in this population, and we expect the population to be heterogeneous with respect to the types of common mental disorders, we aimed for a relatively conservative estimate of a 50 % reduction in HADS overall score in the PM+ group as compared to a 30 % reduction in the control group at 3 months follow-up (this estimate was extrapolated from a trial involving lay workers in India) [24]. This corresponds with an odds ratio for the PM+ program of 2.3. Power calculations suggest a minimum sample size of 133 participants per group (power = 0.95, alpha = 0.05, two-sided). Taking into account an expected 30 % attrition at 3 months follow-up, we aim to include a total number of 346 participants (173 in the PM+ group and 173 in the TAU control group).

### Randomisation

Randomisation will occur following pre-assessment. This will be conducted by an independent research assistant located off-site (Human Development Research Foundation, Islamabad, Pakistan) and not involved in the delivery of PM+, clinical supervision, assessments or other aspects of the day-to-day running of the study. Randomisation will be performed using computerized software on a 1:1 basis. Participants randomised into the PM+ condition will be allocated to a PM+ lay-counsellor by the same independent researcher who performed the randomisation. This lay-counsellor will plan five consecutive meetings with the participant, with the first session being scheduled no longer than 1 week after the pre-intervention assessment.

### PM+

Developed by WHO, PM+ is a new, brief, psychological intervention program based on established problem-solving and behavioural therapy techniques [9]. The program consists of 5 weekly sessions lasting 90 min. Session one orients participants to the program with motivational interviewing techniques to improve engagement, provides psychoeducation about common reactions to adversity, and teaches participants a basic stress management strategy. The latter strategy is practiced at the conclusion of every subsequent session to enhance learning. Session two addresses a participant-selected problem through the provision of problem solving techniques and introduces commencement of behavioural activation procedures. Sessions three and four continue to support participants’ application of problem solving, behavioural activation, and relaxation exercises, and introduces strategies to strengthen social support networks. In session five, education about retaining treatment gains is given, all learned strategies are reviewed, and the program is finished. For a more detailed description of the development of the manual, see Dawson et al. [9].

PM+ providers are male and female lay-counsellors who will have received 8 days of training in basic counselling skills and delivery of PM+ (inclusive of 6-day initial training and a 2-day refresher training before the start of the trial). Weekly supervision will be provided by three supervisors who have received an additional 1-day intensive training in supervisory techniques. In addition, supervisors will receive monthly supervision with the PM+ master trainer, a clinical psychologist (KD), to ensure treatment adherence and provide support. Treatment fidelity will be ensured by an independent assessor who has been trained in PM+. Independent assessors will also directly observe a sample of 10 % of lay-counsellor’s sessions and will systematically assess which elements of the program have been carried out by the PM+ provider using a checklist.

### Treatment-as-usual (TAU)

TAU in primary healthcare centres in Peshawar for common mental disorders usually consists of no treatment, or a range of alternate treatment regimes, such as vitamin injections; evidence-based mental health care is currently not available in PHCs. For this study, participants in the TAU condition will be referred to their primary care physicians for treatment. These primary care physicians will receive the standard training in treatment of common mental disorders that is routinely taught by the Lady Reading Hospital. If, during this treatment or during the study’s assessments participants in TAU arm show severe psychiatric disorders (e.g., psychosis) or problems (e.g., imminent suicidality) that require immediate specialist treatment and follow-up, they will be referred to a psychiatrist within the Lady Reading Hospital.

Throughout the study, we will carefully keep track of the types and amount of support participants receive through the SRI instrument (see measures section below).

### Screening measures

Risk of suicide and presence of severe psychiatric symptoms as defined by the International Statistical Classification of Diseases and Related Health Problems-10th revision (ICD-10) [25] will be assessed using the assessment tools consistent with the mhGAP Intervention Guide [14].

The WHODAS [13] is a generic assessment instrument assessing health and disability. Simple to administer, it is applicable across all health states, including mental disorders, and across cultures. The WHODAS assesses difficulties people have due to their illness across six domains of functioning (cognition, mobility, self-care, getting along, life activities, and participation). Difficulties are scored over the last 30 days on a five-point Likert scale as none, mild, moderate, severe, or extreme. The 12-item interviewer administered version will be used in this study.

The GHQ-12 [11, 12] assesses level of general psychological distress during screening. It consists of 12 questions that are scored on a four-point Likert scale ranging from 0 to 3. When used as a screening tool, the GHQ-12 is usually scored bi-modally (i.e.,-0-0-1-1)., and the total score ranges between 0 and 12, with higher scores representing higher levels of distress. In previous studies in Pakistan, cut-offs of 1 or higher and 2 or higher have been reported and used for determining clinical caseness of common mental health disorders [12, 26, 27].

### Primary outcomes

In addition to the WHODAS, psychological distress in terms of states of anxiety and depression is measured using the HADS [16, 17]. The HADS is a well-established 14-item scale consisting of two sub-scales: HADS-A (anxiety, seven items, range 0–21) and HADS-D (depression, seven items, range 0–21). Higher scores indicate more anxiety and/or depression. The Urdu version of the HADS showed satisfactory reliability and validity [17].

### Secondary outcomes

The PHQ-9 is nine-item instrument measuring presence and severity of depression during the past 2 weeks [18]. The PHQ-9 questions are derived from the 16-item version. Participants rate their responses on a four-point Likert scale ranging from “not at all” to “nearly every day”. The the PHQ-9 total severity score ranges from 0 to 27. The PHQ has been validated in Urdu [28, 29].

DSM-5 posttraumatic stress disorder (PTSD) symptoms during the past week will be measured using the PCL-5 [21], which is a 20-item checklist. Items are rated on a 0–4 scale and add up to a total severity score of 80. The previous version of the PCL based on DSM IV PTSD symptoms, the PCL-C, has been used previously in Pakistan [30] and has been found to have acceptable psychometric properties (Mushtaq, unpublished data, 2013). The PCL-5 will be adapted to ask for symptoms in the last week (rather than month) to enhance sensitivity to change.

Finally, PSYCHLOPS [22] assesses progress on problems for which the person seeks help. It consists of four questions that encompass three domains: problems (2 questions), functioning (1 question) and wellbeing (1 question). Participants are asked to give free text responses to the problem and function domains. Responses are scored on an ordinal six-point scale producing a maximum score of 18 (six points per domain). The PSYCHLOP version administered at posttreatment and follow-up also includes an overall valuation question (determining self-rated outcome ranging from “much better” to “much worse”). PSYCHLOPS has been validated in primary care populations across several countries [31, 32].

### Other measures

To assess the experience of potentially traumatic events, part one of the HTQ [19] will be administered. This includes 17 items describing a range of traumatic events, such as: “lack of food and water”, “forced separation from family members”, and “being close to death”. Each event is rated as either present (1) or absent (0). The HTQ has been validated and applied in many countries, including Pakistan [33].

Life events other than potentially traumatic events (e.g., loss of job, housing problems, financial difficulties, problems with the law, marital problems, bereavement, etc.) are assessed using a life events measure previously developed for the Pakistani population (LELFP [20]). Life events are rated as either present (1) or absent (0).

### Economic evaluation

The Service Receipt Inventory (SRI) assesses service utilization during the time preceding the assessment and related characteristics of people with mental disorders, as the basis for calculating the costs of care for mental health cost-effectiveness research [23]. It has been previously used in Pakistan and India [23, 34].

### Adverse events reporting

All adverse reactions and serious adverse events (SAEs) that are reported spontaneously by the participant or observed by the investigators or other staff members during the trial will be recorded by the research team and will be reported to the local independent advisory board. The chair of the advisory board will review SAEs within 48 h and the advisory board will review all AEs twice a month, where necessary determining any appropriate action in respect of ongoing trial conduct. The consent process includes informing participants whom they can contact if they become distressed or are displeased with any aspect of the study.

Depending on the nature of the adverse event, follow up may require additional tests or medical procedures as indicated, and/or referral to the general physician or a medical specialist. All adverse events will be followed until specialist care (including referrals, additional tests or medical procedures) is in place for the client, or until a stable situation has been reached.

The principal investigator will inform the participants and the local independent advisory board if anything occurs, when it appears to the project group that the disadvantages of participation may be significantly greater than was foreseen.

### Analysis plan

To determine comparability between the conditions at baseline, multiple planned comparisons will be conducted for continuous variables and chi-squared tests for categorical ones.

Hierarchical linear modelling (HLM) analysis will be carried out to assess differential change over time in mean HADS anxiety, depression and total score, WHODAS, and PCL scores between groups. For each outcome, the effects of time of measurement, group, and the group-by-time interaction will be analysed. We will add the following covariates at baseline to examine subgroup effects: gender, education, and severity of symptoms. HLM presumes intent-to-treat analyses as HLM allows the number of observations to vary between participants and effectively handles missing data. Time (linear and quadratic), treatment condition, and their interaction will be included in the models. Fixed effects parameters will be tested at 95 % CI. The Level 1 model will represent within-patient change over time, and the Level 2 model will predict variation in within-patient change over time and encompass between-patient variables.

We will also analyse aggregated health care costs, computed from costs of treatment (primary care, outpatient hospital visits, impatient admission, diagnostic tests and investigations, drug prescriptions), and the aggregated patient and family costs (number of days with reduced working hours, informal caregiving time by relatives or friends), and travel costs and time spent travelling to or waiting for consultations. Cost data will also be analysed using HLM, analysing effects of time of measurement, group, and the group-by-time interaction on aggregated health care costs and patient and family costs.

Descriptive analyses will be carried out in SPSS and HLM analyses in Stata version 11.2. Across all analyses, two-tailed tests will be reported with Cronbach alpha = .05.

### Ethics

The project has been approved locally by the Institutional Review and Ethics Board of the Postgraduate Medical Institute, Lady Reading Hospital, Peshawar, Pakistan and by the WHO Ethical Review Committee (Protocol ID: RPC627, January 20, 2015).

## Discussion

This RCT examines the effectiveness and cost-effectiveness of PM+ in Peshawar, Pakistan. PM+ is a brief transdiagnostic psychological intervention delivered by paraprofessionals for people in low-income communities affected by adversity. It aims to fill an urgent need by providing an evidence-based low-intensity mental health intervention that is amenable to LMICs. A second RCT on the effectiveness of individually delivered PM+ is planned to be carried out in women affected by adversities in Nairobi, Kenya. A third RCT on the effectiveness of group delivered PM+ is planned to be carried out Swat, Pakistan. If proven effective, PM+ may not only be used in similar areas in Pakistan, but rolled out to other affected areas for further adaptation and testing in diverse humanitarian settings. The PM+ manual, if proven effective, will be published by WHO and will be made available (with accompanying training materials) for free on WHO’s website.

## References

[1] Khalily MT (2011). Mental health problems in Pakistani society as a consequence of violence and trauma: a case for better integration of care. Int J Integ Care.

[2] Tol WA, Barbui C, Galappatti A, Silove D, Betancourt TS, Souza R (2011). Mental health and psychosocial support in humanitarian settings: linking practice and research. Lancet.

[3] Jafar TH, Haaland BA, Rahman A, Razzak JA, Bilger M, Naghavi M (2013). Non-communicable diseases and injuries in Pakistan: strategic priorities. Lancet.

[4] Whiteford HA, Degenhardt L, Rehm J, Ferrari AJ, Feigin V, Vos T (2013). Global burden of disease attributable to mental and substance use disorders: findings from the Global Burden of Disease Study 2010. Lancet.

[5] Kohn R, Saxena S, Levav I, Saraceno B (2004). The treatment gap in mental health care. Bull World Health Organ.

[6] Cuijpers P, Sijbrandij M, Koole SL, Andersson G, Beekman AT, Reynolds CF (2013). The efficacy of psychotherapy and pharmacotherapy in treating depressive and anxiety disorders: a meta-analysis of direct comparisons. World Psychiatry : Off J World Psych Asso.

[7] Bisson JI, Ehlers A, Matthews R, Pilling S, Richards D, Turner S (2007). Psychological treatments for chronic post-traumatic stress disorder. Systematic review and meta-analysis. Br J Psychiatry: J Mental Sci.

[8] Patel V, Chowdhary N, Rahman A, Verdeli H (2011). Improving access to psychological treatments: lessons from developing countries. Behav Res Ther.

[9] Dawson KB, Bryant RA, Harper M, Kuowei T, Rahman A, Schafer A, et al. Problem Management Plus (PM+): A WHO transdiagnostic psychological intervention for common mental health problems. submitted. 2015, in press. 10.1002/wps.20255 PMC4592660 26407793

[10] Applied Mental Health Research Group (2013). Design, implementation, monitoring, and evaluation of mental health and psychosocial assistance programs for trauma survivors in low resource countries; a user’s manual for researchers and program implementers.

[11] Goldberg DW P (1988). A user’s guide to the general health questionnaire.

[12] Minhas FM, Mubbashar MH (1996). Validation of general health questionnaire (GHQ-12) in primary care settings of Pakistan. J Coll Physicians Surg Pak.

[13] WHO (2010). Measuring health and disability; manual for WHO disability assessment schedule WHODAS 2.0.

[14] WHO (2010). mhGAP intervention guide for mental, neurological and substance use disorders in non-specialized health settings.

[15] WHO. The process of obtaining informed consent. http://www.who.int/ethics/review-committee/guidelines/en/ (accessed September 4 2013).

[16] Zigmond AS, Snaith RP (1983). The hospital anxiety and depression scale. Acta Psychiatr Scand.

[17] Mumford DB, Tareen IA, Bajwa MA, Bhatti MR, Karim R (1991). The translation and evaluation of an Urdu version of the hospital anxiety and depression scale. Acta Psychiatr Scand.

[18] Kroenke K, Spitzer RL, Williams JB (2001). The PHQ-9: validity of a brief depression severity measure. J Gen Intern Med.

[19] Mollica RF, Caspiyavin Y, Bollini P, Truong T, Tor S, Lavelle J (1992). The harvard trauma questionnaire-validating a cross-cultural instrument for measuring torture, trauma, and posttraumatic-stress-disorder in indo-Chinese refugees. J Nerv Ment Dis.

[20] Rahman A, Iqbal Z, Harrington R (2003). Life events, social support and depression in childbirth: perspectives from a rural community in the developing world. Psychol Med.

[21] Weathers FW, Litz BT, Keane, TM, Palmieri PA, Marx BP, Schnurr PP. The PTSD Checklist for DSM-5 (PCL-5): Scale available at from the National Center for PTSD at http://www.ptsd.va.gov/professional/assessment/adult-sr/ptsd-checklist.asp

[22] Ashworth MS M, Christey J, Matthews V, Wright K, Parmentier H, Robinson S, Godrey E (2013). A client-generated psychometric instrument: the development of “PSYCHLOPS”. Couns Psychother Res: Linking Res Pract.

[23] Chisholm D, Knapp MR, Knudsen HC, Amaddeo F, Gaite L, van Wijngaarden B (2000). Client socio-demographic and service receipt inventory--european version: development of an instrument for international research. EPSILON study 5. European psychiatric services: inputs linked to outcome domains and needs. Br J Psychiatry Suppl.

[24] Patel V, Weiss HA, Chowdhary N, Naik S, Pednekar S, Chatterjee S (2010). Effectiveness of an intervention led by lay health counsellors for depressive and anxiety disorders in primary care in Goa, India (MANAS): a cluster randomised controlled trial. Lancet.

[25] WHO (1994). The ICD-10 classification of mental and behavioural disorders: diagnostic criteria for research.

[26] Shoukat S, Anis M, Kella DK, Qazi F, Samad F, Mir F (2010). Prevalence of mistreatment or belittlement among medical students—a cross sectional survey at a private medical school in Karachi, Pakistan. PLoS One.

[27] Kidwai R (2014). Demographic factors, social problems and material amenities as predictors of psychological distress: a cross-sectional study in Karachi, Pakistan. Soc Psychiatry Psychiatr Epidemiol.

[28] Husain N, Gater R, Tomenson B, Creed F (2006). Comparison of the personal health questionnaire and the self reporting questionnaire in rural Pakistan. JPMA.

[29] Ahmer S, Faruqui RA, Aijaz A (2007). Psychiatric rating scales in Urdu: a systematic review. BMC Psychiatry.

[30] Khalily MTG S, Mushtaq R, Jahangir SF (2012). To examine delayed PTSD symptomatology over time among trauma survivors in Pakistan. Online J Couns Educ.

[31] Czachowski S, Seed P, Schofield P, Ashworth M (2011). Measuring psychological change during cognitive behaviour therapy in primary care: a polish study using ‘PSYCHLOPS’ (psychological outcome profiles). PLoS One.

[32] Heoinsson H, Kristjansdottir H, Olason DP, Sigurosson JF (2013). A validation and replication study of the patient-generated measure PSYCHLOPS on an Icelandic clinical population. Eur J Psychol Assess.

[33] Halepota AA, Wasif SA (2001). Harvard Trauma Questionnaire Urdu translation: the only cross-culturally validated screening instrument for the assessment of trauma and torture and their sequelae. JPMA.

[34] Buttorff C, Hock RS, Weiss HA, Naik S, Araya R, Kirkwood BR (2012). Economic evaluation of a task-shifting intervention for common mental disorders in India. Bull World Health Organ.

