# Correction to: Problem management plus (PM+) for common mental disorders in a humanitarian setting in Pakistan; study protocol for a randomised controlled trial (RCT)

**DOI:** 10.1186/s12888-018-1922-5

**Published:** 2018-10-15

**Authors:** Marit Sijbrandij, Saeed Farooq, Richard A Bryant, Katie Dawson, Syed Usman Hamdani, Anna Chiumento, Fareed Minhas, Khalid Saeed, Atif Rahman, Mark van Ommeren

**Affiliations:** 1 Department of Clinical Psychology, VU University Amsterdam and EMGO Institute for Health and Care Research, Amsterdam, The Netherlands; 2 Post Graduate Medical Institute, Lady Reading Hospital, Peshawar, Pakistan; 3 School of Psychology, University of New South Wales, Sydney, Australia; 4 University of Liverpool and Human Development Research Foundation, Islamabad, Pakistan; 5 WHO Collaborating Centre for Mental Health Research, Training and Substance Abuse and Institute of Psychiatry, Rawalpindi Medical College, University of Health Sciences, Rawalpindi, Pakistan; 6 WHO Regional Office for the Eastern Mediterranean, Cairo, Egypt; 7 Department of Mental Health and Substance Abuse, World Health Organization (WHO), Geneva, Switzerland

## Correction

Following publication of the original article [1], the first author reported an error in referring a paper. This correction presents the incorrect, as well as the correct reference.

Incorrect reference:

- Ashworth MS M, Christey J, Matthews V, Wright K, Parmentier H, Robinson S, et al. A client-generated psychometric instrument: the development of “PSYCHLOPS”. Couns Psychother Res: Linking Res Pract. 2013;4(4):27–31.

Correct reference:

- Ashworth M, Shepherd M, Christey J, Matthews V, Wright K, Parmentier H, Robinson S, Godfrey E. A client-centred psychometric instrument: the development of ‘PSYCHLOPS’ (‘Psychological Outcome Profiles’). Counselling and Psychotherapy Res 2004;4:27–33.
